# Multifunctional Carbon Nanostructures for Advanced Energy Storage Applications

**DOI:** 10.3390/nano5020755

**Published:** 2015-05-08

**Authors:** Yiran Wang, Huige Wei, Yang Lu, Suying Wei, Evan K. Wujcik, Zhanhu Guo

**Affiliations:** 1 Integrated Composites Laboratory (ICL), Department of Chemical & Biomolecular Engineering, University of Tennessee, Knoxville, TN 37976, USA; E-Mails: ywang160@utk.edu (Y.W.); hwei@lamar.edu (H.W.); 2 Materials Engineering and Nanosensor Laboratory (MEAN), Dan F. Smith Department of Chemical Engineering, Lamar University, Beaumont, TX 77710, USA; E-Mail: ylu2@lamar.edu; 3 Department of Chemistry and Biochemistry, Lamar University, Beaumont, TX 77710, USA

**Keywords:** multifunctional, nanocarbon, nanocomposite, nanomaterial, energy storage, capacitor, battery, fuel cell, graphene, CNT

## Abstract

Carbon nanostructures—including graphene, fullerenes, *etc.*—have found applications in a number of areas synergistically with a number of other materials.These multifunctional carbon nanostructures have recently attracted tremendous interest for energy storage applications due to their large aspect ratios, specific surface areas, and electrical conductivity. This succinct review aims to report on the recent advances in energy storage applications involving these multifunctional carbon nanostructures. The advanced design and testing of multifunctional carbon nanostructures for energy storage applications—specifically, electrochemical capacitors, lithium ion batteries, and fuel cells—are emphasized with comprehensive examples.

## 1. Introduction

Sustainable energy from renewable sources—such as wind, hydroelectric, geothermal, biological, nuclear, and solar—are in urgent demand due to the ever increasing energy crisis arising from the limited reserves of fossil fuels [1,2,3,4]. Given the intermittent nature of these renewable energy resources, reliable energy storage systems are critically needed to store and supply energy in a stable manner. Electrochemical energy storage systems (EESS)—including electrochemical capacitors (ECs), batteries, and fuel cells—join various existing energy storage systems such as pumped hydro storage, thermal energy storage, compressed air energy storage, and flywheel energy storage [5,6]. Carbon nanostructures and their multifunctional composites have attracted significant research interest for EESS applications due to their large specific surface area and excellent electrical conductivities [7,8,9,10,11].

Carbon nanomaterials including Buckminster fullerenes [12,13,14], carbon nanotubes (CNTs) [15,16,17,18,19], and graphene [20,21,22,23,24] have attracted much attention in the last three decades. Their outstanding thermal [25,26], electrical [27,28,29,30], optical [31,32,33], and mechanical [26,34] properties, as well as large aspect ratios and higher specific surface areas [35,36], are promising for applications including sensors [37,38,39,40,41,42,43,44], photovoltaics [45,46,47], field emission transistors [48,49,50], fuel cells [51,52,53], supercapacitors [54,55,56,57], composites [58,59,60,61,62], biomaterials [63,64], and environment remediation [65,66], among others. On the nanoscale, the physical and chemical behaviors of the nanomaterials are strongly determined by their structure and interfacial interactions with their surrounding bulk materials. Each type of carbon materials has its own pros and cons. For example, CNTs is a good choice to increase the energy density of ECs considering their unique tubular porous structure and superior electrical properties. However, the high production costs largely limit more widespread applications [67]. Graphene, as a 2D carbon nanostructure, has the advantages of high surface area and high conductivity but it is easy to get restacked during the preparation [61]. Three-dimensional hierarchical carbon materials like activated carbon and templated carbon are highlighted due to their high surface area and rich pore structures. However, the specific capacitance is largely limited at high current density due to the presence of micropores and relative low conductivity [68]. Onion-like carbon can be fully accessible to ions, resulting in excellent performance but limited capacitance (~30 F·g^−1^) [69]. Due to recent developments in the synthesis and manipulation of carbon nanomaterials—including controlled synthesis [70,71,72], functionalization [73,74], and self-assembly [75,76] techniques—and their multifunctional composites, energy storage performances have appreciably increased [77,78,79,80]. Previous reviews regarding these multifunctional carbon nanostructures (MCNs) have been published elsewhere [81,82,83,84,85]; however, very few are focused on recent energy storage applications. Herein, the progress of the many recent advances in energy applications involving MCNs will be discussed. This shall be approached with an emphasis on energy storage applications in three distinct areas: electrochemical capacitors, lithium ion batteries (LIBs), and fuel cells.

## 2. MCNs for Electrochemical Capacitors

ECs have been found to be promising for bridging batteries (high energy density but relatively low power density) and conventional capacitors (high power density but low energy density) with their relatively high both power and energy densities, exceptional long cycling life, and reliability [86,87]. Carbon nanostructures including activated carbons (ACs), carbon nanotubes (CNTs), carbon nanofibers (CNFs), carbon onions, and graphene have been utilized to fabricate electric double layer capacitors (EDLC) due to their high specific surface area and high electrical conductivity [88,89,90]. The energy is stored via the physical adsorption/desorption of ions at the electrode/electrolyte interface, which is a non-Faradic process where no oxidation/reduction reactions are involved. The energy storage mechanism allows a fast charge/discharge rate, *i.e.*, high power density (>10 kW·kg^−1^) and superb cycling stability (>10^6^ cycles). However, their energy density is still lower than those of pseudocapacitors where charges are stored or released through the redox reactions occurring at or near the surface of the electrode material [91,92].

**Figure 1:**
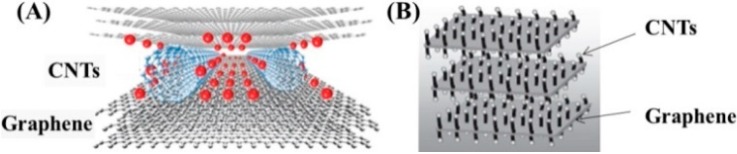
Two models of the 3D CNTs/graphene sandwich structures, where CNTs serve as a spacer to stabilize graphene sheets from agglomeration. Adapted from [97] with permission from the PCCP Owner Societies and from [98] with permission from John Wiley and Sons, 2010.

To increase the energy densities of carbon nanostructures, different strategies have been explored, *i.e.*, to increase the effective utilization of the specific surface area or to introduce pseudocapacitance to the electrode material [93]. For example, considerable research interest has been focused on the full utilization of the surface area of graphene. These materials have been proposed as the next generation of electrode materials for EDLCs due to their high surface area (a theoretical value of 2630 m^2^·g^−1^), sufficient porosity, superior electrical conductivity, broad potential window, and rich surface chemistry [56]. The oxidation and subsequent reduction of graphene oxide (GO) has proved to be the most effective method in the mass production of graphene for industrial applications [94,95]. Unfortunately, reduced GO is prone to agglomeration due to the strong van der Waals interactions between graphene sheets, which ultimately results in unsatisfactory supercapacitive performances [96]. Combining graphene with other carbon nanomaterials such as CNTs has been explored. As illustrated in Figure 1, CNTs were grown in between the graphene sheets and distributed uniformly, but sparsely, on the entirety of the sheets’ surface to form 3D CNT/graphene sandwich structures [97,98]. Herein, CNTs function as “spacers” to stabilize the graphene from aggregation so that the entire graphene surface can be accessed by electrolyte ions.

**Figure 2:**
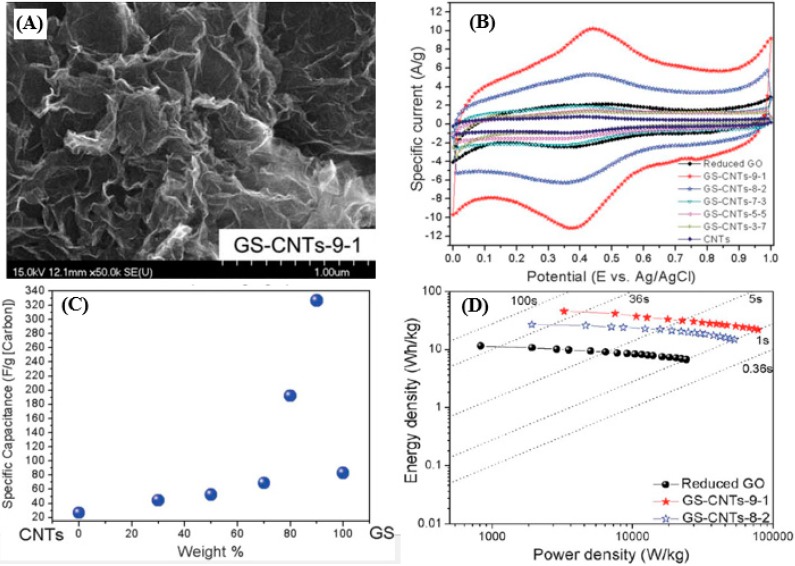
(**A**) FE-SEM image of GS-CNTs-9-1 (where the weight feed ratio of GO to MWNTs is 9:1). (**B**) CV curves measured at 20 mV·s^−1^ in 0.5 M H_2_SO_4_ for GS-CNTs nanocomposites with various weight ratios of reduced GO to MWNTs. (**C**) The dependence of *Cs* on the composition for GS-CNTs nanocomposites. (**D**) A plot of energy density against power density for reduced GO and GS-CNTs nanocomposites. Reproduced from [99] with permission from the Royal Society of Chemistry, 2011.

Yang *et al.* [99] designed a simple route to enhance the utilization of graphene nanosheets (GS) and to improve the capacitive performances through combining 1D CNTs and 2D GS. The effects of the microstructure and capacitive performance of the GS-CNT composites are systematically investigated by varying the weight ratio of reduced graphene oxide (rGO) to MWNTs. Figure 2a shows the FE-SEM image of GS-CNTs-9-1 (where the weight feed ratio of GO to MWNTs is 9:1), which exhibits a more crumpled and rougher surface compared to other GS-CNTs. The stacking of GS is largely inhibited due to the inset of small amounts of MWCNTs. Yet excess MWNTs gave rise to typical MWNTs morphology and GS agglomerates. The GS-CNTs-9-1 also possesses the largest enclosed CV area at a scan rate of 20 mV s^−1^ (Figure 2b), indicating the highest energy storage capability. The corresponding calculated specific capacitance is 326.5 F/g, much higher than the GS-CNTs with other weight ratios (Figure 2c), suggesting the most effective utilization of GS in the composites. In the Ragone plot (Figure 2d), the GS-CNTs-9-1 is observed to deliver an energy density of 45.3 Wh·kg^−1^ with a power density of 3.3 kW·kg^−1^ at a long current drain time (50 s) in a three-electrode setup, much higher than that of rGO (11.5 Wh·kg^−1^ and 0.8 kW·kg^−1^). This shows the promise of the 3D hierarchical GS-CNTs nanocomposites for high-power EC applications with high energy densities.

**Figure 3:**
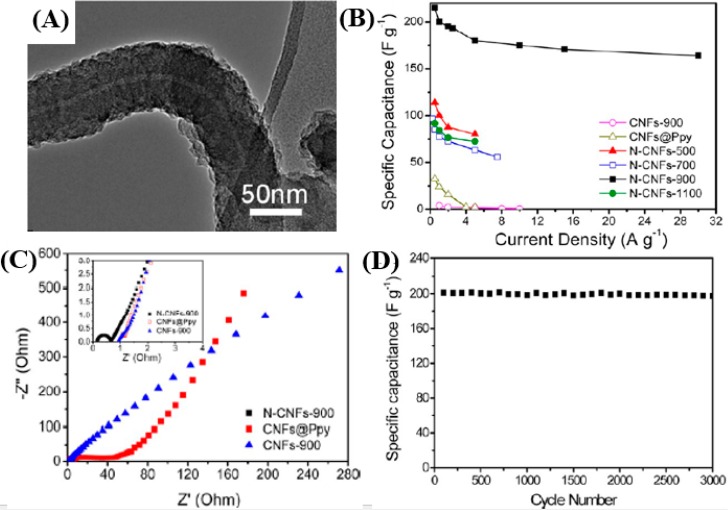
(**A**) TEM images of N-doped CNFs annealing at 900 °C. (**B**) Specific capacitances of CNFs-900, CNFs@Ppy, N-CNFs-500, N-CNFs-700, N-CNFs-900, and N-CNFs-1100 at varying current densities. (**C**) Electrochemical impedance spectra (inset: magnified 0–4 Ω region) under the influence of an AC voltage of 5 mV. (**D**) Cycling performance of the N-CNFs-900 as gravimetric capacitance calculated from the discharge curves of 3000 cycles. Reproduced from [103] with permission from American Chemical Society, 2012.

Another effective strategy to enhance the energy density of carbon nanostructure-based ECs is to introduce pseudocapacitance to the EDLCs. Two approaches have been developed. The first one is to introduce heteroatoms into the carbon frameworks [100,101]. Certain nitrogen doping approaches appear to be the most promising methods so far, while maintaining superb cycling stability [102]. Chen *et al.* [103] reported a facile route to prepare high-content nitrogen-doped porous CNFs containing graphitic frameworks by using macroscopic-scale CNFs as the precursor. Polypyrrole (PPy) was polymerized onto CNFs followed by carbonization at different temperatures (500, 700, 900, and 1100 °C) in an N_2_ atmosphere to obtain a high content of nitrogen doping in CNTs. The X-ray photoelectron spectroscopy (XPS) and N_2_ adsorption measurements reveal that the nitrogen-doped CNFs annealed at 900 °C (N-CNFs-900) demonstrated localized graphitic structure and more favorable morphologies featured with a larger specific surface area and interconnected pores. The high-resolution TEM (HRTEM) image of the N-CNFs-900 is shown in Figure 3a. As theorized, the N-CNFs-900 displays higher specific capacitances at varying charge/discharge current densities than its counterparts in a three-electrode configuration, Figure 3b. An excellent rate capability of 81.72% is also obtained at higher current densities (>1 A·g^−1^). The Nyquist impedance spectrum, Figure 3c, further discloses a smaller semicircle, characteristic of a lower charge transfer resistance, in the high frequency region in the N-CNFs-900 electrode. The equivalent serial resistance (ESR) for the N-CNFs-900 is very low, about 0.14 Ω, compared to 1.25 Ω for the CNFs@Ppy and 0.92 Ω for the CNFs-900. The lower ESR is crucial for achieving higher rate capability and power density for the electrochemical capacitors. A good cycling stability is also obtained in the N-CNFs-900 electrode by retaining above 97% of the initial capacitance after 3000 cycles (Figure 3d).

**Figure 4:**
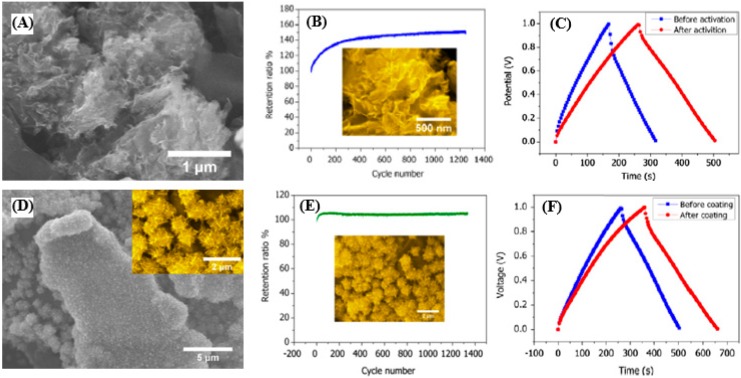
(**A**) SEM image of grapheme. (**B**) Electro-activation of graphene electrode; the SEM image below the curve shows the morphology of the graphene after activation. (**C**) Comparison of charge and discharge curves before and after electro-activation. (**D**) SEM image of MnO_2_-coated grapheme; the inset is a part of the image highlighting the MnO_2_ nanostructures. (**E**) Capacitance retention of graphene electrode after MnO_2_ coating; the image below the retention curve shows the SEM morphology after cycling. (**F**) Comparison of charge and discharge curves before and after MnO_2_ coating. Reproduced from [112] with permission from Elsevier, 2011.

The second approach to introduce pseudocapacitance into carbon is to fabricate carbon-based pseudocapacitive composites [67,104]. For example, transition metal oxides, intrinsically conductive polymers including polyaniline (PANI) [105,106,107,108], polypyrrole (PPy) [109,110], and polythiophene or their derivatives [111] are commonly used as pseudocapacitive electrode materials with high specific capacitances due to their rich redox reactions. Cheng *et al.* [112] fabricated graphene/MnO_2_ composite electrodes by *in situ* anodic electrodeposition of MnO_2_ on the graphene electrode (apparent electrode surface area is 2 cm^2^). The electrochemical properties of the pristine graphene and the composites were examined in 1 M KCl using a two-electrode configuration. Figure 4a depicts the SEM image of the graphene, which exhibits wrinkled-paper-like morphologies. The pure graphene paper shows an enhanced capacitance under 4 mA charge/discharge rate for 1300 cycles (Figure 4b). The long time charging and discharging may help the ions to fully access to the graphene surface area, as confirmed by the increased capacitance. A 60% increase in the capacitance is observed in Figure 4c, further confirming the positive role of the electro-activation process, which is probably due to the movement of GS in order to adjust to the different electrolyte ions. The morphology of the graphene coated with MnO_2_ flowers is shown in Figure 4d. The MnO_2_ nanoflowers are composed of many tiny nanorods (the inset of Figure 4d). The retention ratio in Figure 4e remains almost constant in the first 150 cycles, and only dropped by 1% after 1300 cycles, indicating an excellent cycling stability of the MnO_2_-coated graphene. The charging and discharging curves under 1 mA of the electrode before and after MnO_2_ coating are shown in Figure 4f. The discharge time becomes longer and the corresponding calculated specific capacitance increases from 245 to 328 F/g after the MnO_2_ coating. Correspondingly, the energy density increases from 8.5 to 11.4 Wh·kg^−1^.

**Figure 5:**
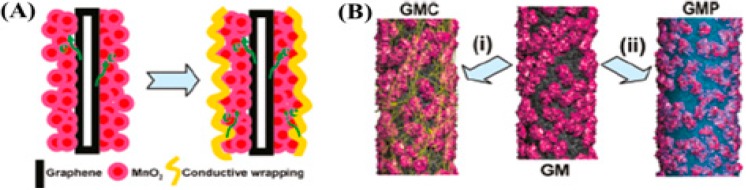
(**A**) Schematic illustration showing conductive wrapping of graphene/MnO_2_ (GM) to introduce an additional electron transport path (in a discharge cycle). (**B**) Models of graphene/MnO_2_/CNT (GMC) and graphene/MnO_2_/conducting polymer (GMP) systems formed by wrapping of GM nanostructures with CNTs or conducting polymers. Reproduced from [114] with permission from the American Chemical Society, 2011.

However, even though an enhanced capacitance and energy density have been achieved after the introduction of metal oxides to the carbon nanostructures, there are still concerns about the poor electrical conductivity of the metal oxide coating [113]. Decreased supercapacitive performances are expected in practical applications where high mass loading of the electrode materials are densely packed to achieve the desired capacitance and energy storage. To address this problem, Yu *et al.* [114] have developed a “three dimensional (3D) conductive wrapping” method to rationally design ternary systems. Using graphene as a model carbon material, they have prepared graphene/MnO_2_/CNT (GMC) and graphene/MnO_2_/poly(3,4-ethylenedioxythiophene) poly-(styrenesulfonate) (PEDOT:PSS) (GMP) composites for the high-performance electrochemical electrodes (Figure 5).

Besides the graphene layer underneath the MnO_2_, the ultrathin layer of SWNTs or conducting polymer that wraps around graphene/MnO_2_ (GM) three-dimensionally can also provide an additional electron transport path. Simultaneously, the SWNTs and polymer are actively participated in the charge storage process since both can contribute to the whole capacitance via electric double layer capacitance or pseudocapacitance. As expected, GMP and GMC exhibit superior performances in terms of capacitance performance and rate performance in comparison with GM. Although the GMP system offers higher specific capacitance than the GMC, due to large pseudocapacitance contributed from the conductive polymer, the wrapping with CNTs can be advantageous in those applications that require high operation voltages owing to their better electrochemical stability across a large voltage range and environmental safety.

The combination of different carbon materials renders a greatly enhanced surface area and improved chemical stability, thus favoring the EC performance. Furthermore, the introduction of pseudocapacitive heteroatoms and organic materials also offers a good way forward to design multifunctional carbon nanostructures with high stability, excellent functionality, and moderate cost, which are prerequisites for scaleable ECs.

## 3. MCNs for Lithium Ion Batteries

Carbon-based rechargeable batteries have gained extensive attention, particularly after the commercialization of the Li-ion battery (LIB) from Sony laboratories. Here, the metallic lithium is replaced by a carbon host structure, which can reversibly absorb/desorb lithium ions at low electrochemical potentials [115,116,117,118]. Yet, there are disadvantages associated with carbon-based anode materials—for example, low theoretical specific capacity (372 mAh·g^−1^ for graphite [119]) and low rate capability.

**Figure 6:**
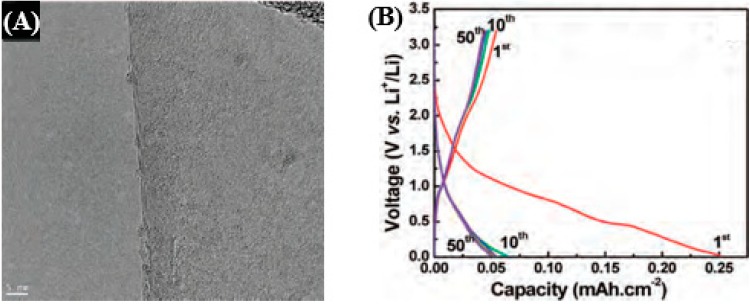

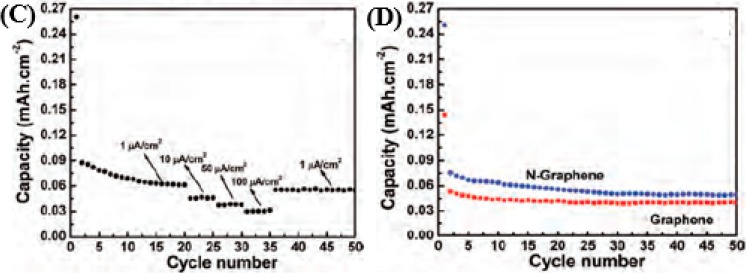
(**A**) HRTEM image of three-layered N-doped grapheme. (**B**) Charge-discharge voltage profiles for the N-doped graphene electrode cycled at a rate of 5 μA·cm^−2^ between 3.2 and 0.02 V *vs.* Li/Li^+^. (**C**) Rate capability studies of N-doped graphene films: discharge capacity *vs.* cycle number at various current rates (1, 10, 50, and 100 μA·cm^−2^). (**D**) Cycling stability for the pristine graphene and N-doped graphene cycled at a rate of 5 μA·cm^−2^ between 3.2 and 0.02 V *vs.* Li/Li^+^. Reproduced from [121] with permission from the American Chemical Society, 2010.

To improve their performances, considerable efforts have been focused on surface modification by chemical doping [120]. Chemical dopants such as phosphorus, boron, and nitrogen have been reported to enhance the Li-ion storage capability originating from the enhanced reactivity and electric conductivity. For example, Reddy *et al.* [121] demonstrated a controlled growth of nitrogen-doped graphene layers by liquid precursor-based chemical vapor deposition (CVD) technique and evaluated its applications for LIBs. Figure 6a shows the high-resolution TEM (HRTEM) micrograph of the three-layered N-doped grapheme, where both amorphous carbon and crystalline graphene were observed. Figure 6b shows the voltage *versus* specific capacity plots conducted at 5 μA·cm^−2^ between 3.2 and 0.02 V *vs* Li/Li^+^ in 1 M LiPF6 in a 1:1 (v/v) mixture of ethylene carbonate (EC) and dimethyl carbonate (DMC). The first cycle shows a discharge capacity of around 0.25 mAh·cm^−2^ and a loss in the capacity is observed in the second cycle due to the solid electrolyte interface (SEI) formation. Figure 6c shows the rate capability study of the N-doped graphene electrode. Even operated at very high current rates, *i.e.*, 100 μA·cm^−2^, excellent capacity retention of 60% of the nominal capacity was observed, indicating that the N-doped graphene electrode could be used as an excellent high rate electrode for LIB. A comparison of the cycling stability is also conducted at 5 μA·cm^−2^ between 3.2 and 0.02 V *vs.* Li/Li^+^ as shown in Figure 6d. The N-doped graphene shows higher discharge capacity (0.05 mAh·cm^−2^) than that of pristine graphene (0.03 mAh·cm^−2^) and remains stable over 50 cycles. The increase in the reversible discharge capacity of the N-doped graphene over pristine graphene can be attributed to the topological defects induced in the N-doped graphene electrode. First, a large number of surface defects are induced onto the graphene films by N-doping, which leads to the formation of a disordered carbon structure that further enhanced the Li^+^ intercalation abilities. The N-doped graphene also has a high percentage of pyridinic N atoms, which could also be involved in the improved reversible capacity of the N-doped graphene electrode compared to that of the pristine graphene electrode.

The doping effect on other carbon nanostructure as anodes for LIB has also been studied using porous carbon nanofiber webs (CNFWs). For example, Qie *et al.* [53] reported a facile strategy to synthesize CNFWs using a PPy precursor synthesized via a modified oxidative template assembly route, followed by carbonization-activation with KOH.

**Figure 7:**
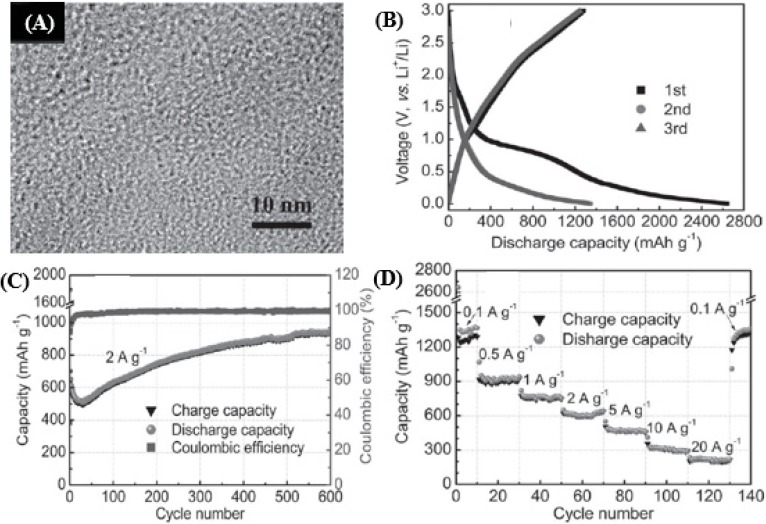
(**A**) HRTEM of carbon nanofiber webs (CNFWs) doped with nitrogen. (**B**) Charge/discharge curves at 0.1 A/g. (**C**) Cyclability and Coulombic efficiency at 2 A/g; and (**D**) capacity over cycling at different rates. Reproduced from [53] with permission from John Wiley and Sons, 2012.

Figure 7a shows a HRTEM micrograph of the CNFWs. Large quantities of micropores are uniformly distributed within the CNFWs. The uniform micropores are inferred to result from the combustion of PPy precursors during the pyrolysis process. Figure 7b shows the charge/discharge profiles of the CNFWs during the initial three cycles at a current density of 0.1 A·g^−1^. The initial reversible capacity can reach as high as 1280 mAh·g^−1^, more than three times higher than the theoretical value of graphite (372 mAh·g^−1^). The CNFWs’ anode also displays excellent cycling performance with high capacities (Figure 7c). The reversible capacity is 633 mAh·g^−1^ in the first cycle and then gradually increases to 943 mAh·g^−1^ after 600 cycles. The increase in the capacity with the cycling can be attributed to the activation of the porous anode. Such an exceptionally high Li^+^ ion storage capacity is claimed to be first witnessed in a carbon-based anode material. It is worth noting that the CNFWs anode still shows high Li ^+^ ion storage and excellent cycling stability even at very high rates (Figure 7d). For testing, the cell was first discharged/charged at a current density of 0.1 A·g^−1^ for 10 cycles, and then at various current densities ranging from 0.5 to 20 A·g^−1^ each for 20 cycles. The reversible capacities are 924, 773, 637, 505, and 321 mAh·g^−1^ at 0.5, 1, 2, 5, and 10 A·g^−1^, respectively. Even at an extremely high current density of 20 A·g^−1^ (~40 s to full charge), the reversible capacity is 226 mAh·g^−1^, which is about 60% of the theoretical capacity of graphite. When the rate is switched back to 0.1 A g^−1^ after cycling at different rates, the specific capacity can be recovered to 1321 mAh·g^−1^, indicating an excellent cycling performance. Apart from the carbon-based anodes, the transitional metal oxides including Co_3_O_4_, MoO_3_, and Fe_3_O_4_ are another important type of anode material for LIB [122,123,124]. These transitional metal oxides can react with 6 Li^+^ per formula unit, giving rise to a significantly larger reversible capacity than that of commercial graphite. However, large volume expansion/contraction during the Li^+^ insertion/extraction and serious particle aggregation leading to a serious loss of capacity with cycling and a poor cycling stability [125]. Meanwhile, carbon-based anode materials, especially graphene, are known for their large initial discharge capacity and high reversible capacity [126]. Therefore, the combination of nanostructures with transitional metal oxides to achieve desirable electrochemical performance has proved to be an effective strategy [127,128].

**Figure 8:**
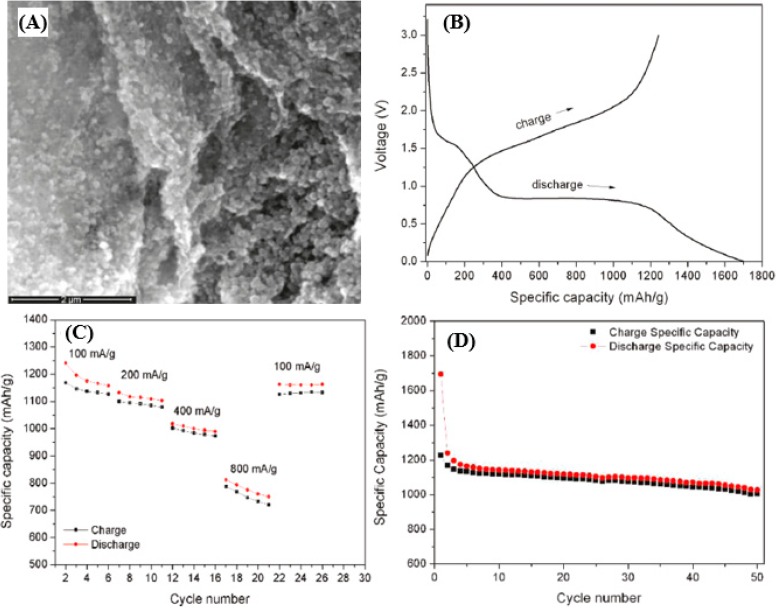
(**A**) SEM image of rGO/Fe_2_O_3_ composites. (**B**) First discharge/charge profiles of rGO/Fe_2_O_3_ composites for the first cycle at the current density of 100 mA/g. (**C**) Rate capacity of rGO/Fe_2_O_3_ composites between 0.05 and 3.0 V with different current densities. (**D**) Cycling performance of rGO/Fe_2_O_3_ composites at the current density of 100 mA/g. All the specific capacities are based on the mass of Fe_2_O_3_. Reproduced from [129] with permission from the American Chemical Society, 2011.

The reduced graphene oxide (rGO)/Fe_2_O_3_ composites for LIB anode materials were reported by Zhu *et al.* [129], employing a facile two-step synthesis method. Figure 8a presents an SEM image of the rGO/Fe_2_O_3_ composite. The Fe_2_O_3_ NPs are observed to be uniformly distributed on the surface of rGO, confirming the successful synthesis of rGO/Fe_2_O_3_ composites. The rGO/Fe_2_O_3_ composites exhibit two obvious plateaus (~1.6 and ~0.8 V *vs.* Li^+^/Li) in the initial discharge/charge curve due to the lithium reaction with Fe_2_O_3_ nanoparticles (Figure 8b). The discharge and charge capacities are 1693 and 1227 mAh·g^−1^, respectively, based on the mass of Fe_2_O_3_. An excellent rate performance is also accomplished in rGO/Fe_2_O_3_ composites, which can reach as high as ~800 mAh·g^−1^ even at a current density of 800 mA·g^−1^ (Figure 8c). Figure 8d shows the cycling performance of the rGO/Fe_2_O_3_ composites at 100 mAh·g^−1^ in the range between 3.0 and 0.005 V. The superior capacity and good capacity retention observed in the rGO/Fe_2_O_3_ composites are explained by the Fe_2_O_3_ nanoparticles anchored on the surface of the rGO templates, which behave as spacers and greatly facilitate the lithium insertion/extraction.

In summary, porous nanostructures can not only shorten the transport length for Li^+^ but also offer a large electrode/electrolyte interface for the charge-transfer reaction. Chemical dopants in carbon materials such as phosphorus, boron, and nitrogen can modify the carbon structure, thus contributing to a substantial increase in specific capacity relative to pure carbon due to the enhanced reactivity, electric conductivity, and Li^+^-ion storage capacity. Incorporating metal oxides in carbon nanostructures is also another alternative owing to the high theoretical capacity and capability of intake excess of Li^+^ ions of metal oxides during the charge-discharge process.

## 4. MCNs for Fuel Cells

A third important application for MCNs is for the oxygen reduction reaction (ORR) in the fuel cells to replace the Pt-based catalysts, which are costly and have limited availability and poor durability [130]. Both experimental and theoretical studies show the possibility of making p- and n-type semiconductors by substituting C atoms with B and N atoms in the carbon frameworks in graphene and CNTs. The doped atoms are capable of modifying the electronic band structure of the carbon nanostructures, and consequently tuning the mechanical properties and electrocatalytic activity.

**Figure 9:**
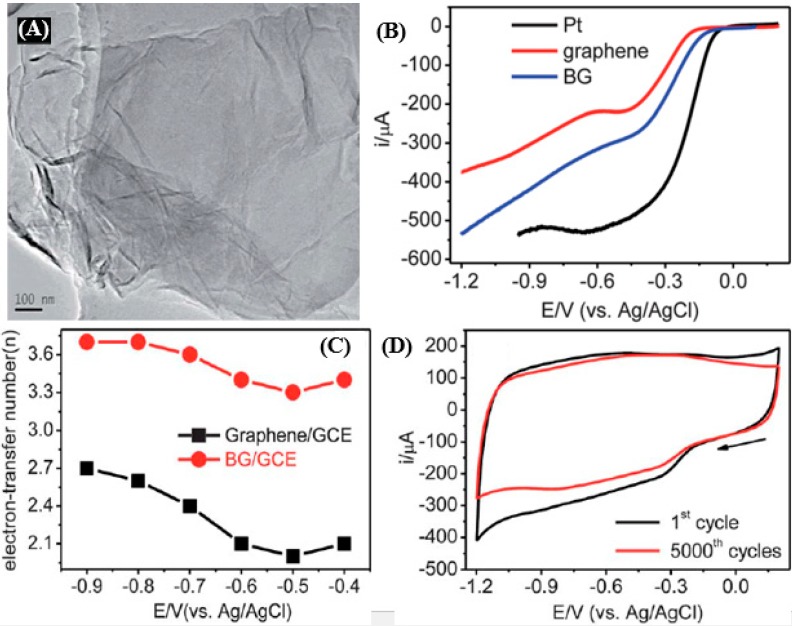
(**A**) TEM image of boron-doped graphene (BG). (**B**) Liner sweep voltammetric curves (LSVs) of ORR for graphene/GCE (red), BG/GCE (blue) and bulk Pt disk electrode (black) in an O_2_-saturated 0.1 M KOH aqueous solution (scan rate: 10 mV·s-1). The rotation rate of RDE was 1200 rpm. (**C**) The dependence of n on potential for the BG/GCE and pure graphene/GCE. (**D**) CVs of BG/GCE for ORR in O_2_-saturated 0.1 M KOH aqueous solution. The first scan (black) and the 5000th scan (red). Scan rate: 100 mV·s^−1^. Reproduced from [131] with permission from the Royal Society of Chemistry, 2012.

For example, Sheng *et al.* [131] reported a facile and catalyst-free approach to synthesize boron doped graphene (BG) by thermal annealing GO in the presence of B_2_O_3_. The electrocatalytic performance was evaluated in a three-electrode setup using BG or a pristine graphene-modified glassy carbon electrode (denoted as BG/GCE and graphene/GCE, respectively) in an O_2_-saturated 0.1 M KOH aqueous solution. Figure 9a shows the TEM image of BG, where the flattened BG nanosheets are randomly stacked together with a flake-like structure similar to that of the pristine graphene. Figure 9b shows the linear sweep voltammetric curves (LSVs) of ORR at graphene/GCE, BG/GCE, and a bulk Pt disk electrode at a scan rate of 10 mV·s^−1^. The onset potentials for ORR at pristine graphene are at ~−0.15 and ~0.59 V, respectively. This indicates that the ORR process catalyzed by the pristine graphene is a two-step, two-electron pathway with the formation of intermediate HO_2_^−^ ions. In contrast, the BG/GCE, in the case of the Pt electrode, exhibits a one-step process for ORR with the onset potential at about −0.05 V, which is about 100 mV more positive than that on the pristine graphene/GCE. The one-step process suggests a four-electron pathway for the ORR at BG. Both the positive shift of the onset potential and the enhanced reduction current for ORR on BG/GCE demonstrate that BG possesses much higher electrocatalytic activity towards ORR than graphene, which could be explained by the faster reaction kinetics with a higher transferred electron number per oxygen molecule at the former (Figure 9c). The average electron transfer number *n* for ORR at BG/GCE is calculated as 3.5 over the potential range from −0.4 to −0.9 V, which is much higher than that of the pristine graphene (2.1–2.7), indicating that the N-doped carbon materials demonstrate much better electrocatalytic activity towards ORR than pristine carbon. The BG catalyst shows considerable stability during ORR after 5000 cycles (Figure 9d), indicating an excellent electrocatalytic performance of BG after the incorporation of B atoms into graphene. Recently, researchers have also found that graphene doped with elements that have similar electronegativities to carbon, such as sulfur and selenium, can also exhibit better catalytic activity than commercial Pt/C catalyst in alkaline media [132]. This study gives further insight into the ORR mechanism of these metal-free doped carbon materials, but also opens ways to fabricate other new low-cost non-precious-metal catalysts. However, these doped carbon materials usually exhibit ORR activity in alkaline electrolytes but poor activity in acids.

**Figure 10:**
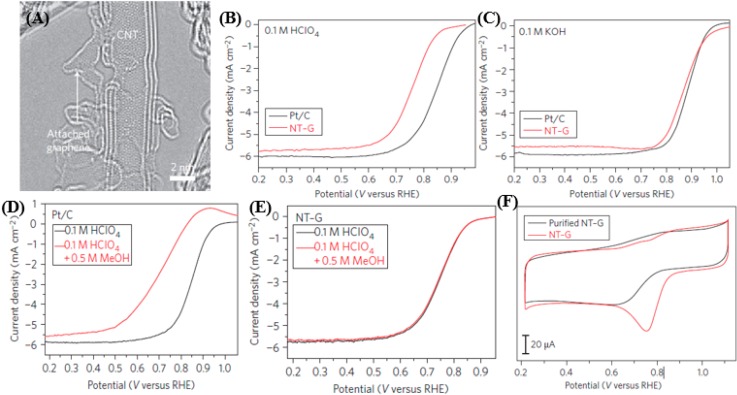
(**A**) Aberration-corrected TEM images of the NT-G (carbon nanotube–graphene complex) material, showing damaged outer walls and exfoliated graphene pieces attached to double- or triple-walled carbon nanotubes (CNTs). RRDE polarization curves of 20% Pt/C (black) and NT-G (red) in (**B**) O_2_-saturated 0.1 M HClO_4_ and (**C**) O_2_-saturated 0.1 M KOH, respectively. (**D**,**E**) RRDE polarization curves of the NT-G catalyst before and after 8000 potential cycles in argon- or O_2_-saturated 0.1 M HClO_4_, respectively. Potential cycling was carried out between 0.6 and 1.0 V *versus* RHE at 50 mV/s. (**F**) Comparison of the RRDE polarization curves of NT-G with/without intentional iron impurity removal. Reproduced from [133] with permission from Nature Publishing Group, 2012.

To address this problem, Li *et al.* [133] fabricated few-walled carbon nanotube-graphene (NT-G) complexes, which show ORR activities in both acidic and alkaline solutions. Figure 10a presents high-resolution aberration-corrected transmission electron microscopy (TEM) images of the NT–G material. The nanotubes are mostly two- to three- walled, with their outer walls damaged and exfoliated to form single-layered nanosized graphene pieces (~5 nm) or ribbon-like structures by the harsh oxidation process. These graphene structures exhibit abundant edges and are often found attached to the nanotubes. Regions with intact inner walls on the exfoliated nanotubes are also frequently observed. Figure 10b,c depicts the comparisons of ORR catalytic activity on 20 wt% Pt on Vulcan carbon black (Pt/C from E-tek) and NT-G in acidic and basic media, respectively. The catalyst shows markedly higher ORR catalytic activity than previous CNT-based electrocatalysts in the acidic medium of 0.1-M HClO_4_. In 0.1-M KOH electrolyte, the NT-G catalyst shows an ORR activity closely approaching that of the Pt/C. Figure 10d,e show the rotating ring disk electrode (RRDE) polarization curves measured in the presence of 0.5-M methanol. A drastic decrease in the ORR activity of Pt/C is observed. In contrast, the NT-G catalyst shows little activity loss, exhibiting excellent tolerance to methanol poisoning. Figure 10f shows the CV curves of NT—before and after purification of iron. The purification and iron removal steps cause a large loss in ORR activity in the purified NT-G, with an onset-ORR potential ~100 mV more negative than NT-G, which indicates a positive role of the iron impurities. Similarly, the advantages of heteroatom doping in carbon structure and the expansion of functional groups have been demonstrated due to the increased active sites, which provide an inexpensive alternatives to precious-metal electrocatalysts in fuel cells.

## 5. Conclusions and Perspectives

This review gives the most recent developments in the field of energy storage devices—specifically, electrochemical capacitors, lithium ion batteries, and fuel cells—utilizing MCNs. On the nanoscale, the physical and chemical behaviors of such MCNs are strongly influenced by their structure and interfacial interactions with their surrounding bulk materials or other nanomaterials. The advanced design and testing of these MCNs for energy storage devices are emphasized with comprehensive examples. There are still some shortcomings and disadvantages related to different carbon nanostructures, such as the irreversible capacity loss, big voltage crosstalk, low density, and so on. Novel composites containing multifunctional nanostructured-carbon and other dopants can synergistically take advantage of the combination of ordered building block units with other desired properties. It is possible to expect a breakthrough improvement of the performance of electrical storage devices if we keep moving forward in this systematic manner accompanied with fundamental understanding of theory and *in situ* experimentation.
